# Eye-color and Type-2 diabetes phenotype prediction from genotype data using deep learning methods

**DOI:** 10.1186/s12859-021-04077-9

**Published:** 2021-04-19

**Authors:** Muhammad Muneeb, Andreas Henschel

**Affiliations:** Department of Electrical Engineering and Computer Science, Khalifa University of Science and Technology, Abu Dhabi, United Arab Emirates

**Keywords:** Bioinformatics, Genotype–phenotype, Eye color, Type-2 diabetes, Machine learning

## Abstract

**Background:**

Genotype–phenotype predictions are of great importance in genetics. These predictions can help to find genetic mutations causing variations in human beings. There are many approaches for finding the association which can be broadly categorized into two classes, statistical techniques, and machine learning. Statistical techniques are good for finding the actual SNPs causing variation where Machine Learning techniques are good where we just want to classify the people into different categories. In this article, we examined the Eye-color and Type-2 diabetes phenotype. The proposed technique is a hybrid approach consisting of some parts from statistical techniques and remaining from Machine learning.

**Results:**

The main dataset for Eye-color phenotype consists of 806 people. 404 people have Blue-Green eyes where 402 people have Brown eyes. After preprocessing we generated 8 different datasets, containing different numbers of SNPs, using the mutation difference and thresholding at individual SNP. We calculated three types of mutation at each SNP no mutation, partial mutation, and full mutation. After that data is transformed for machine learning algorithms. We used about 9 classifiers, RandomForest, Extreme Gradient boosting, ANN, LSTM, GRU, BILSTM, 1DCNN, ensembles of ANN, and ensembles of LSTM which gave the best accuracy of 0.91, 0.9286, 0.945, 0.94, 0.94, 0.92, 0.95, and 0.96% respectively. Stacked ensembles of LSTM outperformed other algorithms for 1560 SNPs with an overall accuracy of 0.96, AUC = 0.98 for brown eyes, and AUC = 0.97 for Blue-Green eyes. The main dataset for Type-2 diabetes consists of 107 people where 30 people are classified as cases and 74 people as controls. We used different linear threshold to find the optimal number of SNPs for classification. The final model gave an accuracy of 0.97%.

**Conclusion:**

Genotype–phenotype predictions are very useful especially in forensic. These predictions can help to identify SNP variant association with traits and diseases. Given more datasets, machine learning model predictions can be increased. Moreover, the non-linearity in the Machine learning model and the combination of SNPs Mutations while training the model increases the prediction. We considered binary classification problems but the proposed approach can be extended to multi-class classification.

## Background

All humans are different from each other like our eye color and other physical characteristics. Why are we all different? Why our physical characteristics differ? The answer to this question lies in genetic variations in human beings [1]. Human DNA consists of about 3 billion bases, and more than 99.9% of those bases are the same in all people [2]. In human DNA, there is only a 0.1% difference. Some genes function as protein-making instructions, and others do not [3]. Genes in humans range in number from a few hundred bases of DNA to more than two million bases. In all humans, most genes are the same, although a limited number of genes within individuals are slightly distinct. Alleles are forms of minor variations of the same gene in their sequence of DNA bases. These small differences contribute to each person’s distinctive physical characteristics [4]. Two kinds of alleles are available. The dominant allele is always expressed, even if there is only one copy of it for the organism. Only if the person has two copies of it and does not have the dominant allele of that gene is a recessive allele expressed. This pair of alleles is known as a genotype and determines the appearance or phenotype of the organism [5].

These physical characteristics may be the product of one gene mutation, such as the Mendelian trait, or more than one gene. If more than one gene mutation affects any physical characteristic, then it is called phenotypes. In humans, most of the traits are polygenic [6]. SNPs are single base pair polymorphic DNA regions that differ from person to person frequently. In each chromosome, SNPs occur with a proportion of 1 SNP per 1000 base pairs [7]. To establish the relationship between genotypes and phenotypes, genome-wide association studies are used and include scanning the genome to identify single nucleotide polymorphisms associated with the phenotype of interest. Positive ties between an SNP and a phenotype mean that the associated SNP contributes to the trait or is similar to a genetic variant in a chromosomal region that contributes to the trait [8].

There are several ways to discover the relation between SNPs and phenotypes. Some are statistical methods and others are approaches to machine learning [9, 10]. This article focus on a hybrid approach that uses thresholding at individual SNP based on mutation and machine learning for finding an association.

The two methods used for Genome-wide-association-studies are quantitative trait locus mapping and Haplotype association. A quantitative trait locus (QTL) is a region of DNA that is linked to a particular phenotype that varies in degree and may be due to polygenic effects. Analysis of variance called marker regression at the marker loci is the simplest approach for QTL mapping. A t-statistic is determined in this method to compare the averages of the two marker genotype groups. The F-statistic is used for more than two potential genotypes [11]. There are various approaches, including score checks, logistic regression, and Bayesian methods, for Haplotype association with a phenotype. Furthermore, both techniques estimate haplotype frequencies since haplotypes are typically not observed directly. If an association signal is detected, it is possible to use linkage imbalance to optimize the signal where an association is detected. Alleles are in linkage disequilibrium when they do not occur spontaneously with respect to each other. If two alleles occur more frequently than expected on the same haplotype, there is a positive linkage imbalance, and negative LD occurs less often than expected when alleles occur together on the same haplotype [10]. A collection of advanced statistical and computational algorithms such as a Support vector machine or Random forest can be used to make predictions by mathematically mapping the complex associations between a set of SNPs to complex phenotypes [12]. To map the associations with the phenotype, these methods use supervised or unsupervised approaches. Prediction models of supervised machine learning traits are developed by training preset learning algorithms to map the relationships between the genotype data of the individual sample and the associated phenotype. By mapping the pattern of the selected characteristics within the training genotype data, optimal predictive capacity for the target phenotype is achieved. Some models use gradient descent procedures and parameter estimation iterative rounds to look for optimal predictive power across the training data space. Machine learning algorithms use multivariate, non-parametric methods that identify patterns from data that are not normally distributed and highly correlated [13, 14].

To predict the phenotype, we can use SNPs. Some Genotype–Phenotype predictions fall into the issue of classification. We used the Machine Learning and Deep Learning methods in this article to find a connection between SNPs and the eye-color phenotype.

We started our research work with a question. “Can deep learning methods outperform existing techniques like Machine learning and Statistical technique?”. To make a proper comparison we must implement the existing techniques, or we must have results from other researchers. We adopted the second approach of using existing results for Eye-color and Type-2 diabetes. There are some common steps before genotype data is passed to deep learning methods like data cleaning, SNPs encoding, etc [15, 16]. After that, we proposed and implemented a simple pipeline which is explained in the manuscript to achieve the existing accuracy with deep learning. Now the question is raised “if we already have methods for phenotype prediction then why use deep learning methods?”

These are the reasons.

- SNPs identified by existing approaches for classification can be different from those identified by the deep learning algorithms, which carry high explanatory value in non-linear decision-making processes and in turn increases prediction accuracy.
- LSTMs [17] and 1D-CNNs [18] are known to perform well at handling sequential data like (text sequences). Genotype data can be treated as sequential data so the information obtained from SNPs in any chromosome can be used for final prediction.
- Deep Learning methods lend themselves to transfer learning [19], which facilitates the transfer of knowledge from large datasets to smaller ones.

Table 1 summarizes the results of already developed techniques. Researchers used Multinomial regression and the Irisplex model for eye color prediction. Evaluation measure is Area under the curve and accuracy shown in the second last column. Last column shows the number of SNPs condsidered for classification.

**Table 1:** Result of eye-color prediction using the already developed techniques for different population

Method	Blue-eyes	Brown-eyes	Population	Metric	SNPs
Multinomiallogistic regression	0.91	0.93	Dutch Europeans [20]	AUC	24
Multinomial logistic regression	–	0.93	Saudi population [21]	AUC	5
IrisPlex model	0.96	0.96	Dutch Europeans [11]	AUC	–
IrisPlex model	0.79	0.91	Iraqi population [22]	AUC	6
Multinomial regression	0.966	0.913	Slovenian population [23]	AUC	6
Decision tree models	0.89	0.94	New Zealand population [24]	Accuracy	6
IrisPlex	0.95	0.58	United States population [25]	Accuracy	6

“–” means no data found for that cell. The last column shows the number of SNPs considered for classification. The second and third column represents the result for Blue eyes and Brown eyes respectively

Type-2 diabetes is a purely polygenic phenotype and finding the optimal number of SNPs can significantly affect the performance of the model. In 2017, about 462 million people were affected by type 2 diabetes which corresponds to 6.28% of the world’s population [26]. Type-2 diabetes is not only related to genotype but there are also many factors like gender, age, Body mass index, and other environmental effects which can affect the risk of developing this particular disease [27].

To classify people into cases or controls rather than using genotype data most of the researchers used different factors like gender, age, body mass index, environmental effects, daily routine, and food consumption [28–30].

**Fig. 1:**
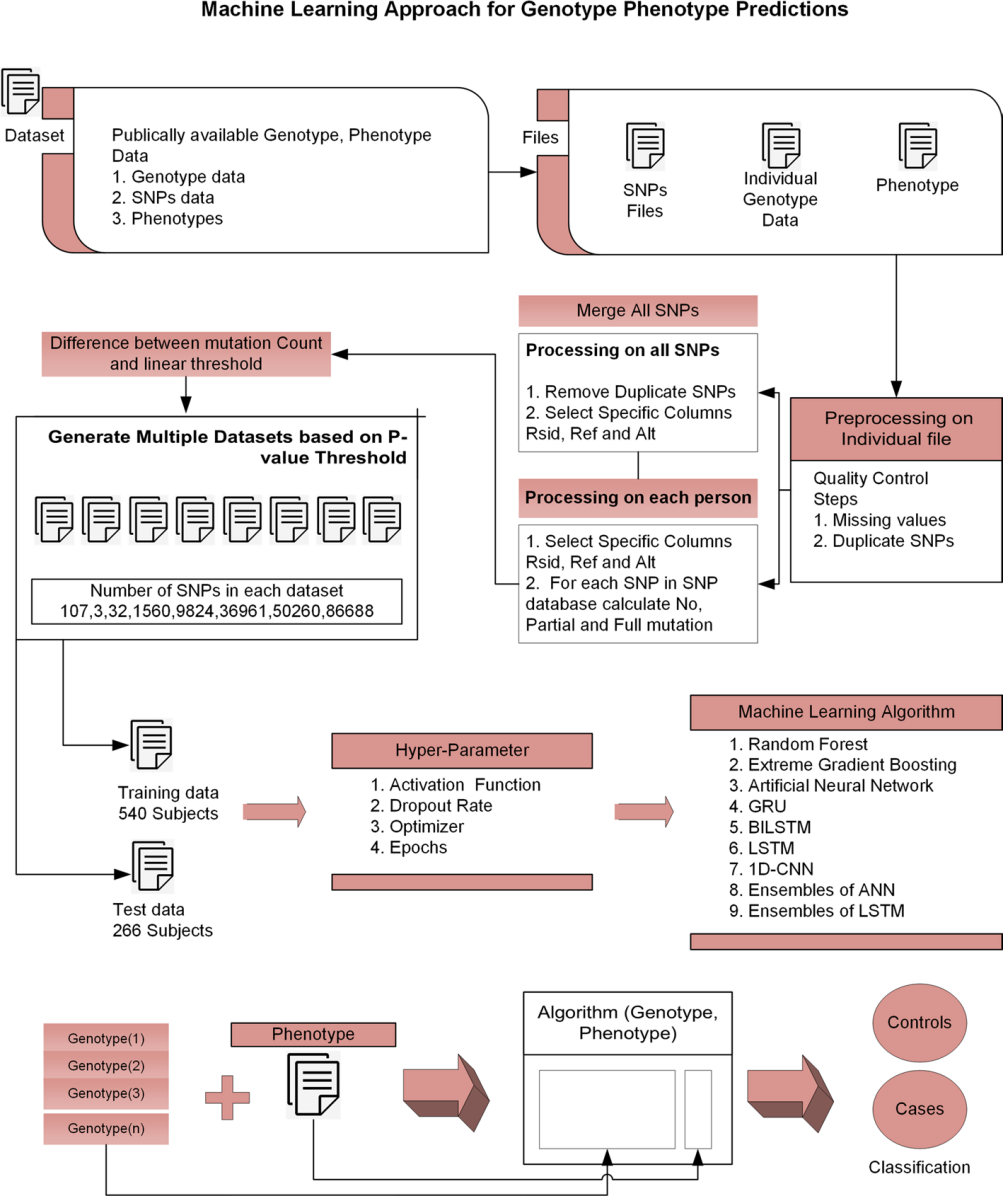
Flowchart of machine learning approach for genotype phenotype predictions. This flowchart presents an overview of the hybrid approach for genotype–phenotype prediction. After cleaning data, multiple datasets were generated using mutation thresholding, containing different numbers of SNPs. Different machine learning algorithms with various hyper-parameters were considered for training the model

Researchers used 408 SNPs in 87 genes involved in significant Type-2 diabetes in 462 cases and 456 Korean cohort studies controls in article [31]. By using the help vector machine strategy, they got a 0.65% accuracy with a combination of 14 SNPs in 12 genes. Figure 1 shows the flowchart of the overall approach.

## Materials and methods

This section summarizes the dataset, methods, and machine learning model used for analysis. There are two main processes in the whole manuscript. The first is the SNPs selection process and the other is finding the best model for classification. There is variation in both processes which can affect the results.

- SNPs pre-selection (number of SNPs to be included or passed to algorithm for classification)
- Finding the best model (variation in hyper-parameters in each model)

To find the best model we designed a simple pipeline to achieve high accuracy. For eye-color phenotype, we generated multiple datasets containing a different number of SNPs. After that, we applied 9 classifiers for each dataset. When we applied an algorithm to any dataset, we also considered different hyperparameters specific to that algorithm to find the optimal result. This whole pipeline is computationally expensive but reliable because after finding the best model which can be from machine learning or deep learning paradigm, we can use it to classify people based on genotype data into specific phenotype. As far as machine learning algorithms (Random forest and XGBOOST) are concerned no additional modifications are performed. But for deep learning algorithms (ANN, 1DCNN, and LSTM) we tried different architectures with different parameters to find the optimal model.

### Dataset

The dataset considered for this analysis is taken from OPENSNP. The dataset consists of 806 people. 404 people have Blue-Green eyes where 402 people have Brown eyes [32].

### Dataset preparation

The dataset we have is in the AncestryDNA, ftdna-illumina, and 23andme file format. All the genotype files must be converted to 23andme standard format. So the AncestryDNA file must be converted to the 23andme file format. Ftdna-illumina is encrypted file format so we ignored files in that format.

AncestryDNA file has 5 columns.

- rsid
- chromosome
- position
- allele1
- allele2

where as 23andme file has 4 columns.

- rsid
- chromosome
- position
- genotype

allele1 is the reference allele whereas allele2 is the alternative allele. These two columns must be merged like this “allele1allel2” to convert AncestryDNA to 23andme file format. After that all the genotype files in 23andme file format.

### Data pre-processing

The dataset have 3 types of files.

- Phenotype
- Genotype
- SNPs

The phenotype file contains the phenotype for each person. Genotype files contain the genotype information for each person. These files are in the standard format. Where SNPs consist of all the SNPs for which associations are to be tested for eye color phenotypes. These files are in VCF format. All the SNPs are merged in one file to make an SNPs database. Before analysis it is important to make sure that dataset is clean.

#### Quality control

Quality control on GWAS data are delicate pre-processing steps for any genotype–phenotype association analysis, and that they can strongly affect results and biological interpretation [33–35]. All the genotype files and SNPs file must be preprocessed before the SNPs pre-selection process. These are the quality control steps considered for this analysis.

- Missing SNPs
- Duplicate SNPs

SNPs with missing reference allele or alternative allele are simply removed without considering any kind of imputation technique. There is a possibility that the SNPs database contains duplicate SNPs, so duplicate SNPs must be removed from the dataset.

#### Coding

The users are grouped by phenotypes and mutations are identified for each SNP. The mutation encoding is 0 for none, 1 for partial, and 2 for full mutation. Tables 2 3 4 show the corresponding calculation for coding and SNPs preselection process.

Counts the number of mutations the user has for each SNP.

**Table 2:** Mutation type at each SNP

SNP	Rsid	Ref	Alt	User genotype	Mutation coding	Mutation type
1	rs3753834	C	T	CC	0	No mutation
2	rs625149	G	T	GT	1	Partial mutation
3	rs625149	G	A	AA	2	Full mutation

Reference allele is compared to person genotype to find mutation type

**Table 3:** Percentage of 3 types of mutation at each SNP

SNP Rsid	Person1	Person2	…	PersonN	PFM	PNM	PPM
rs82	2	1	…	0	57.142857	14.285714	28.571429
rs85	2	1	…	1	57.142857	0	42.857143
…	…	…	…	…	…	…	…
rs373523829	NaN	NaN	…	NaN	0	0	0

Calculate the percentage of 3 types of mutation for each SNP

*PFM* percentage of full mutation, *PNM* percentage of no mutation, *PPM* percentage of partial mutation

#### SNPs preselection

There were about 1304138 SNPs for both phenotypes. 804482 SNPs removed due to too many missing user observations for both phenotype.

**Table 4:** Compare the 3 types of mutations for 2 phenotype

SNP Rsid	PA-PNM	PA-PPM	PA-PFM	PB-PNM	PB-PPM	PB-PFM
rs82	5.21978	36.538462	58.241758	2.754821	33.057851	64.187328
rs85	5.698006	31.908832	62.393162	6.376812	34.202899	59.42029
…	…	…	…	…	…	…
rs72552726	99.6139	0.3861	0	98.876404	1.123596	0

PA means phenotype A and PB means phenotype B. Phenotype A is Brown eye color and Phenotype B is Blue-Green eye color. In general, if Phenotype A represents the case or the Phenotype B is the control or vice-versa. *PFM* percentage of full mutation, *PNM* percentage of no mutation, *PPM* percentage of partial mutation

To reduce the number of SNPs calculate the absolute difference between each type of mutation for both phenotypes. Equations 1 2 3 show the calculation.

1 $$\begin{aligned}&absoluteFullDifference=phenotype_AFM-phenotype_BFM \end{aligned}$$

2 $$\begin{aligned}&absolutePartialDifference=phenotype_APM-phenotype_BPM \end{aligned}$$

3 $$\begin{aligned}&absoluteNoDifference=phenotype_ANM-phenotype_BNM \end{aligned}$$

Absolute Difference for each type of mutation should be greater than predefined threshold. If absolute difference is less than predefined threshold than discard that SNP.

After that, we find the maximum and minimum for each type of mutation which will be used in further calculations for thresholding. It is a single SNP scanning process that is also used in statistical techniques to find the association. Equations 4 5 6 7 8 9 show the calculation where FM means full mutation, PM means Partial mutation and NM means no mutation.

4 $$\begin{aligned}&maxFullMutation=max(phenotype_AFM,phenotype_BFM) \end{aligned}$$

5 $$\begin{aligned}&minFullMutation=min(phenotype_AFM,phenotype_BFM) \end{aligned}$$

6 $$\begin{aligned}&maxPartialMutation=max(phenotype_APM,phenotype_BPM) \end{aligned}$$

7 $$\begin{aligned}&maxPartialMutation=min(phenotype_APM,phenotype_BPM) \end{aligned}$$

8 $$\begin{aligned}&maxNoMutation=max(phenotype_ANM,phenotype_BNM) \end{aligned}$$

9 $$\begin{aligned}&maxNoMutation=min(phenotype_ANM,phenotype_BNM) \end{aligned}$$

Selects SNPs that have a significant difference in mutation percentage between phenotype groups based on a linear threshold that is modeled after the dominant-recessive disease model. Equations 10 11 12 show the calculation for linear thresholding which is repeated for each type of mutation and if SNP is above the lower threshold for any type of mutation then that particular SNP is selected.

10 $$\begin{aligned}&Threshold\,=\,slope*maxFullMutation +intercept \end{aligned}$$

11 $$\begin{aligned}&LowerThreshold =(1-Threshold/100)*maxFullMutation \end{aligned}$$

12 $$\begin{aligned}&selectedSNPs\,=\,minFullMutation<=LowerThreshold \end{aligned}$$

One important point to notice here is the slope in Eq. 10 which is the controlling factor based on which we can increase or decrease the SNPs. A lower value of Slope will lower the mutation threshold and more SNPs will be selected. Whereas increasing Slope will result in a reduction of SNPs but SNPs with high mutations are selected.

Multiple datasets are generated using linear thresholding. These are the controlling factor values and the corresponding number of SNPs.

- Slope = − 1.14, SNPs = 107
- Slope = − 0.5, SNPs = 3
- Slope = − 1, SNPs = 32
- Slope = − 1.5, SNPs = 1560
- Slope = − 2, SNPs = 9,824
- Slope = − 3, SNPs = 36,961
- Slope = − 3.5, SNPs = 50,260
- Slope = 5, SNPs = 86,688

Association studies are commonly used for GWAS by comparing allele or genotype frequencies between Phenotype A and Phenotype B. Indeed, the most widely used technique is the single SNP scan, consisting of sequentially evaluating each SNP with the null hypothesis of no association. In order to associate SNPs to the phenotype, different tests may be used. In principle, machine learning algorithms should deal with genome-wide SNPs. Datasets with a large number of characteristics, however are subject to the curse of dimensionality. Therefore a more efficient approach involves first reducing the total number of SNPs to a manageable level through a screening process and searching for causal loci among those passing data sets containing different numbers of SNPS.

#### Data separation and score

In order to achieve a similar Brown/Blue-Green ratio on all subsets, samples were randomly permuted. The dataset was then divided into a train dataset (540 samples) and a dataset for research (266 samples). The Brown/Blue-Green ratio was around 1. in both the train and test sets. To test the predictions of the different models, we used accuracy as the score. We used different dropout rates for all the understudy models, in order to prevent over-fitting. We then assessed the models trained on the entire train set on the test set.

### Models and implementation

We used Randomforest and Extreme Gradient Boosting on all the datasets, whereas ANN, 1DCNN, LSTM, GRU, BILSTM, and Ensembles of LSTM/ANN were used on datasets containing 3, 32, 107, and 1560 SNPs.

A very critical step in finding the relationship between SNPs and phenotypes is finding the best machine learning architecture. If a model includes several layers and processing units in each layer, then there is a risk of overfitting the training data with the resulting model. If the model of layers and processing units is reduced, then the resulting model underfits the training data. It is necessary to find the optimal model architecture for any machine learning problem [36].

#### Artificial neural network

An ANN has hundreds or thousands of integrated, artificial neurons called processing units. Input and output units are made up of these processing units. Based on an internal weighting scheme, the input units obtain varying sources and structures of information and the neural network aims to learn from the information provided to generate one output report. ANNs often use a series of learning principles called backpropagation, an abbreviation for backward propagation of error, to refine their performance outputs, just as humans need instructions and instructions to come up with a conclusion or output. An ANN initially goes through a training process where it learns to identify trends in SNPs [37, 38]. The network contrasts its real output generated with what it was intended to achieve the desired output during this controlled process. Using backpropagation, the disparity between all effects is modified.This suggests that the network operates backward, moving from the output unit to the input units to change the weight of its interactions between the units until the lowest possible error is generated by the discrepancy between the real and expected result.

Each dataset is passed to ANN with output labels. The main advantage of using ANN is the non-linearity produced by the activation function. For datasets consisting of 3, 32, 107, and 1560 SNPs we used ANN containing a different number of layers and the different number of processing units in each layer.

We can use any activation function like sigmoid equation 13 and relu equation 14 where *x* represent the input. Equation 15 represents the softmax activation function and different elements of the equation.

13 $$\begin{aligned} S\left( x\right)&= {} \frac{1}{1+e^{-x}} \end{aligned}$$

14 $$\begin{aligned} f\left( x\right)&= {} \left\{ \begin{matrix}\ \ \ 0\ \ \ \ for\ \ \ \ x<0\\ \\ \ \ x\ \ \ \ for\ \ \ \ x\ge 0\\ \end{matrix}\right. \end{aligned}$$

15 $$\begin{aligned} \sigma \left( {\vec{z}}_i\right)&= {} \frac{e^{z_i}}{\sum _{j=1}^{K}e^{z_j}} \\ \vec{z}&= {} Input\ Vector \\ {e}^{z_i}&= {} Standard\ exponential\ function\ for\ input\ vector \\ e^{z_j}&= {} Standard\ exponential\ function\ for\ output\ vector \\ K&= {} Number\ of\ classes\ in\ the\ multi-class\ classifier \end{aligned}$$

Equations 16 17 show the functionality of ANN and explain different parameters used in the equation.

16 $$\begin{aligned}&\alpha _j^l=\sigma \left( \sum _{k}\omega _{jk}^l\alpha _k^{l-1}+b_j^l\right) \\&\quad \sigma \ is\ the\ activation\ function \\&\quad \alpha _j^l\ is\ the\ activation\ of\ the\ j^{th}neuron\ in\ the\ l^{th}layer \\&\quad b_j^l\ is\ the\ bias\ of\ the\ j^{th}neuron\ in\ the\ l^{th}\ layer \\&\quad The\ sum\ is\ over\ all\ neurons\ k\ in\ the\ \left( \ l-1\right) ^{th}\ layer \end{aligned}$$

17 $$\begin{aligned}&\omega _{jk}^l\ denote\ the\ weight\ for\ the\ connection\ from\ the \\&\quad k^{th}\ neuron\ in\ the\ \left( \ l-1\right) ^{th}\ layer\ to\ the \\&\quad j^{th}\ neuron\ in\ the\ l^{th}\ layer \end{aligned}$$

**Fig. 2:**
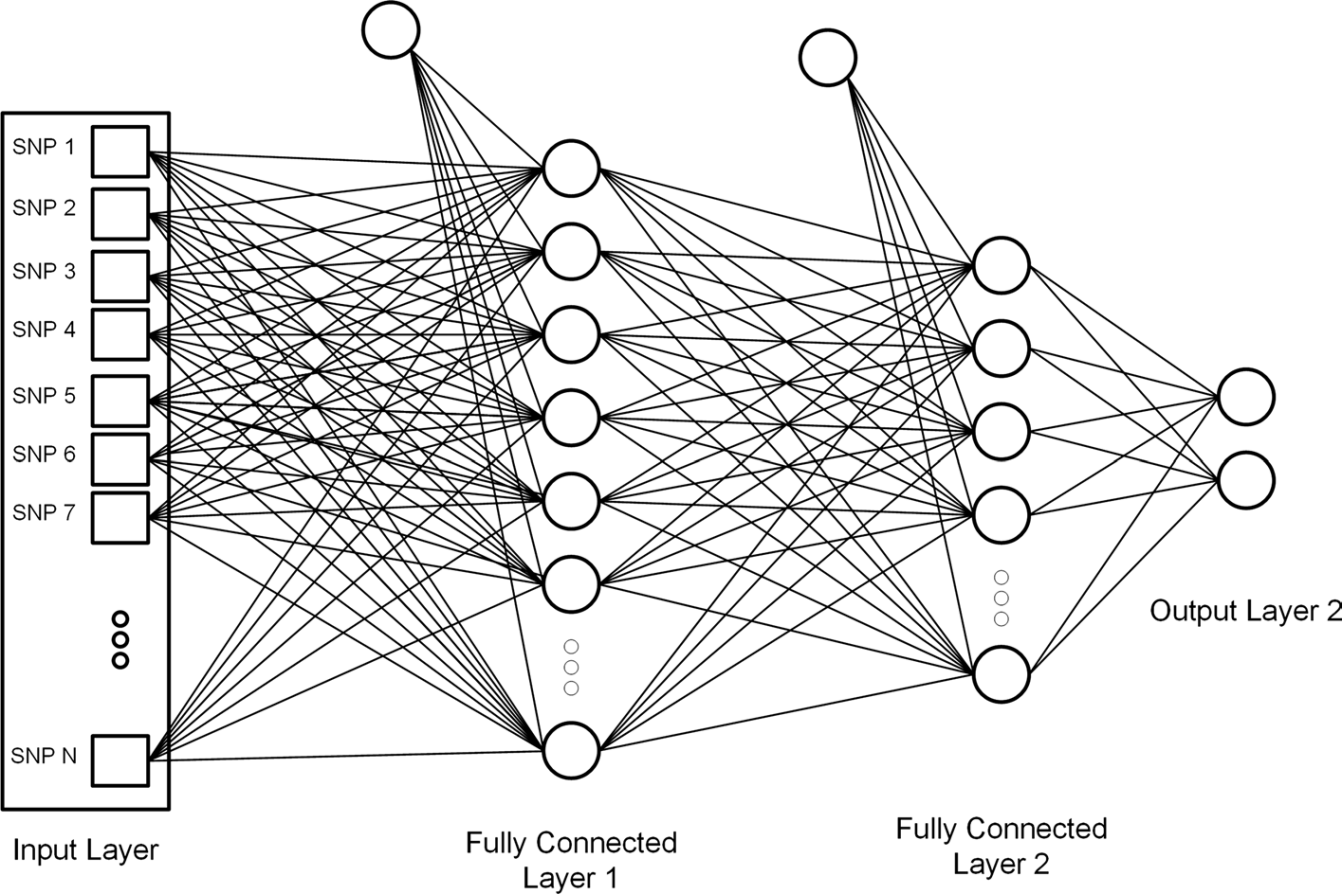
Artificial network network structure. Selected SNPs are passed to a fully connected network. Each connection represents the weight learned by the model. The number of hidden layers and the number of neurons in each layer can be changed. Each circle is a processing unit which will perform will perform activation function on a combination of input from the previous layer. It is a binary classification problem so the output layer contains 2 processing units [39]

Figure 2 shows the architecture of an Artificial Neural Network.

#### One-dimensional convolution neural network

Deep learning is a part of machine learning and can play an important role in real-world applications, such as bioinformatics and computational biology [40], remote sensing [41], photogrammetric computer vision [42], medicine [43], and 3D modeling [44]. Digital signal and image analysis using deep learning methods, particularly convolutional neural networks, is an explosively growing field.

For image recognition, Convolution Neural Network (CNN) models have been generated in which the algorithm accepts a two-dimensional input representing the pixels and color channels of an image, in a process called feature learning. It is possible to extend this same method to one-dimensional data sequences. The model derives characteristics from sequence data and maps the sequence’s internal characteristics. A 1DCNN is very successful in deriving features from the overall dataset’s fixed-length section, where it is not so important where the feature is placed in the segment [16, 45]. Genotype data is sequential information, so it is possible to use 1DCNN for phenotype prediction. This model integrates information from several SNPs and relies on the filter size in each layer on the number of SNPs that would be merged.

**Fig. 3:**
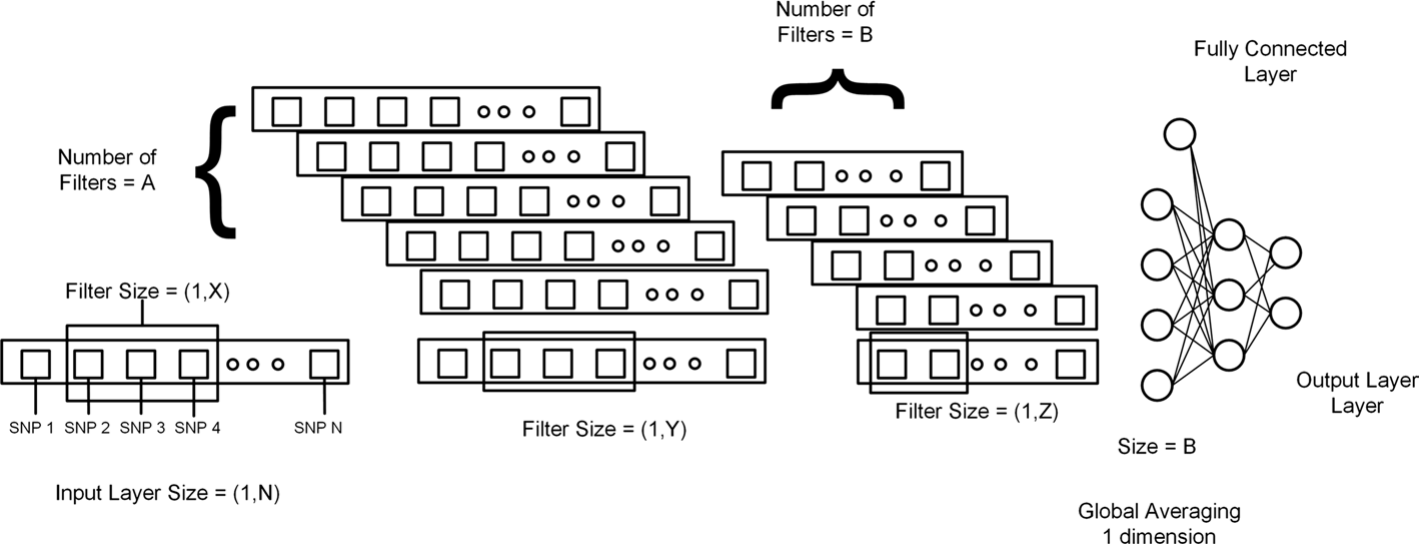
One dimensional architecture. Selected SNPs are passed to a 1DCNN. N, X, Y, and Z represent the size of the input layer, and X, Y, Z represent the filter size for the first layer, second layer, and third layer. A and B represents the number of the filter in the first layer and second layer. As it is 1DCNN so kernel size or filter size has one dimension equal to 1 and the other is variable. The number of hidden layers, the number of filters in each layer, and the size of the filter can be changed. It is important to form the proper model. At the end output of the last 1DCNN layer, after global averaging, is connected to the fully connected network. In a fully connected network number of layers and the number of neurons in each layer can also be changed [45]

Figure 3 shows the architecture of one-dimensional convolution neural network.

#### Recurrent neural network

For sequential data or time-series data, a recurrent neural network (RNN) is used. These are widely used, such as language translation, natural language processing (NLP), voice recognition and image captioning, for ordinal or temporal problems. They identify themselves by their “memory” because they take data from previous inputs to affect the current input and output. Although conventional deep neural networks assume that each other is independent of inputs and outputs, the performance of recurrent neural networks depends on the sequence’s previous elements. Although future events will also help to assess the performance of a sequence in question.

#### Gated recurrent units

Gated recurrent units (GRU) are correlated with LSTM as both use the different way of gating data to avoid the issue of vanishing gradient. The GRU controls, but without having to use a memory unit, the flow of information like the LSTM unit. Without any influence, it only exposes the full secret content. GRUs train faster than LSTMs because fewer parameters are available. It has only two gates, a reset gate and a gate for updates. The update gate works in a similar way to the LSTM forget and input gate. It determines what data to throw away and what fresh data to add. Another gate that is used to determine how much past knowledge to forget is the reset gate.

#### Long short-term memory

As a recurrent neural network, a long short-term memory (LSTM) has a similar control flow. It handles information that passes on data as it propagates forward. The variations are the events inside the cells of the LSTM. Such operations are used to enable the LSTM to retain or forget information. The cell state serves as a network’s “memory”. In principle, the cell state will hold relevant data during the sequence processing. So even data from the earlier time steps will make it possible for later time steps to decrease the short-term memory impact. Data is added or removed to the cell state through gates as the cell state goes on its journey. The gates are different neural networks that decide which knowledge about the cell state is permitted. The gates will learn what data during training is necessary to keep or forget [16].

The Forget Gate decides what information should be thrown away or preserved. The Input Gate is used to change the state of the cell. The gate of production determines what should be the next hidden state. The hidden state includes information about earlier inputs.

The hidden state is also used for predictions. The output is the hidden state. The candidate state is created using combine. The candidate gate holds possible values to add to the cell state. The new cell state and the new hidden cell state are then moved to the next stage. These gates make LSTM suitable for the prediction of genotype–phenotype [46] and we can make few changes to the structure of the LSTM state to make it better for the dataset of genotype.

**Fig. 4:**
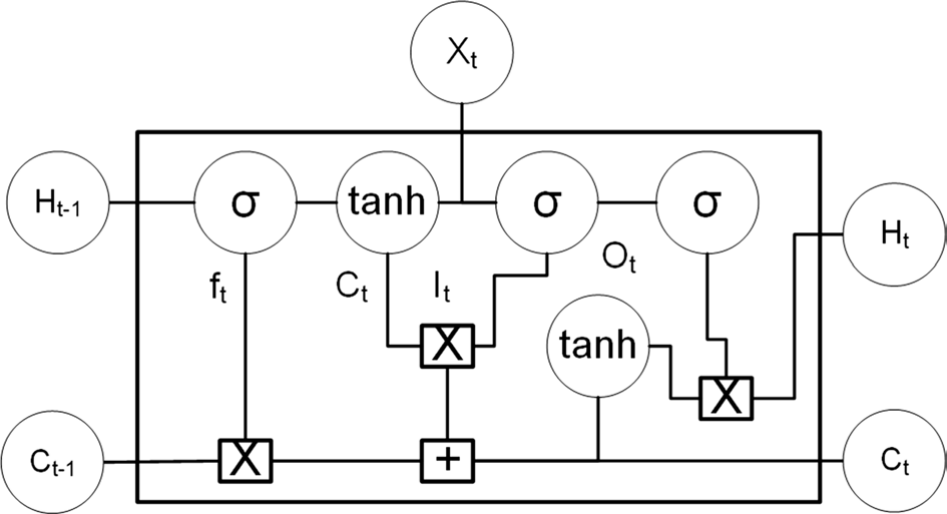
LSTM architecture. Selected SNPs are passed to a LSTM cell

Figure 4 shows the architecture of an LSTM.

Equations 18, 19, 20 represents the functions in LSTM cell. F,C,I and O are the forget, Candidate, Input and Ouput gates.

18 $$\begin{aligned} f_t&= {} sigmoid\left( X_t*U_f+H_{t-1}*W_f\right) \\ {\overline{C}}_t&= {} tanh\left( X_t*U_c+H_{t-1}*W_c\right) \\ f_t&= {} sigmoid\left( X_t*U_i+H_{t-1}*W_i\right) \\ f_t&= {} sigmoid\left( X_o*U_f+H_{t-1}*W_o\right) \\ C_t&= {} f_t*C_{t-1}+I_t*{\overline{C}}_t \\ H_t&= {} O_t*tanh\left( C_{t-1}\right) \end{aligned}$$

19 $$\begin{aligned} X_t&= {} Input\ Vector \\ H_{t-1}&= {} Previous\ Cell\ Output \\ C_{t-1}&= {} Previous\ Cell\ Memory \\ H&_t= {} Current\ Cell\ Output \\ C_t&= {} Current\ Cell\ Memory \end{aligned}$$

20 $$\begin{aligned} W_f,U_f&= {} weight \ vectors\ for\ forget\ gate \\ W_c,U_c&= {} weight\ vectors\ for\ candidate\ gate \\ W_i,U_i&= {} weight\ vectors\ for\ input\ gate \\ W_o,U_o&= {} weight\ vectors\ for\ output\ gate \end{aligned}$$

#### Bidirectional LSTM

A Bidirectional LSTM (BILSTM), is a sequence processing model that consists of two LSTMs: one taking the input in a forward direction, and the other in a backward direction. BILSTMs increase the amount of knowledge accessible to the network efficiently. It involves duplicating the first recurrent layer of the network so that there are two side-by-side layers now then supplying the input sequence to the first layer and providing a reverse copy of the input sequence to the second.

#### Random forest

Random Forest is a combination of many decision trees. A decision tree is a technique for creating classification or regression models. They are called decision trees since many branches of if…then…” decision splits are used for the prediction—similar to the branches of a tree.

The most frequent indicator for determining the best split” is Gini impurity and information gain for classification tasks. Bagging and boosting are two primary ways of integrating the outputs into a random forest of different decision trees.

Bagging, also known as Bootstrap aggregation (used in Random Forests) Bagging works the following way: on randomly sampled subsets of the data, decision trees are trained, while sampling is done with replacement. A major benefit of bagging over individual trees is that the model variance is minimized. Individual trees are very susceptible to overfitting and very sensitive to data noise. As long as our individual trees are not connected, without raising the bias, combining them with bagging will make them more resilient. The final outcome of our model is calculated by averaging over all predictions from these sampled trees or by majority vote [47].

**Fig. 5:**
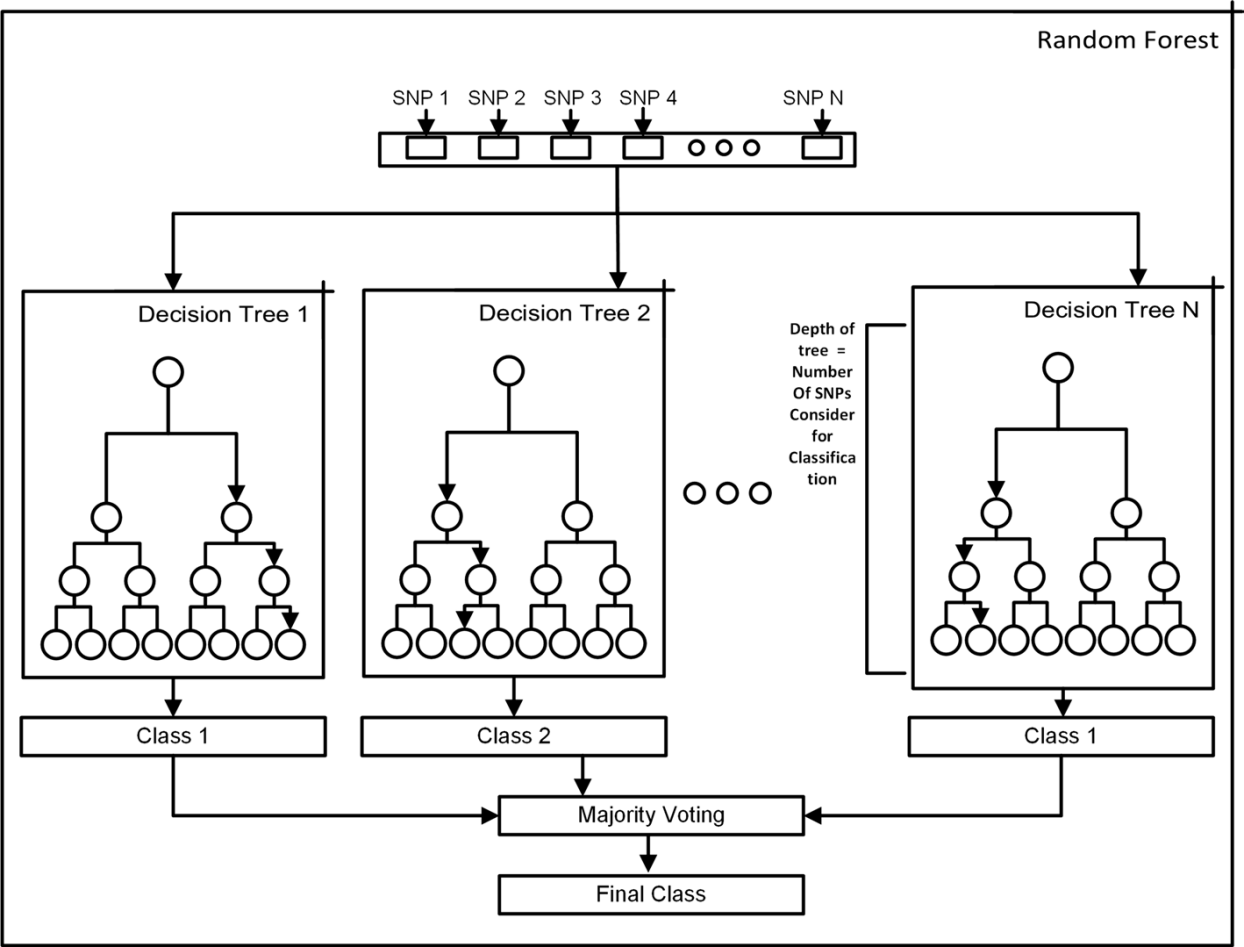
Random forest. Dataset after preprocessing is passed to each Decision Tree. Each decision tree is trained on train data and for each test sample prediction from each decision tree is considered. The final decision for each sample is based on Majority voting. The depth of the tree determines the number of SNPs used for classification. SNPs with high idnformation gain on the top

Random Forest is suitable for genotype data, especially for SNP ranking. They are already used for genotype–phenotype predictions and good at handling noisy data. SNPs that do not contain useful information are discarded and the final prediction is based on the useful SNPs only [48, 49]. Figure 5 shows the working of the random forest.

We used the GridSearch strategy to find the best model. Following are the parameters used in GridSearch.

- criterion =  gini, entropy
- minimum samples split = 0.01, 0.015, 0.02, 0.025
- maximum depth =  None, 4, 5
- minimum samples leaf = 0.0025, 0.005, 0.01, 0.015
- maximum features = sqrt, 0.3, 0.4, 0.5
- number of estimators = 500, 1000, 3000

Figure 5 shows the structure of a Random Forest.

#### XGBOOST

XGBoost stands for Extreme Gradient Boosting; which uses the Gradient Boosting method to find the precise approximations to the best tree model. It employs a range of nifty tricks that make it exceptionally efficient, especially with structured data. In the xgboost model compute the second-order gradients, which offers more knowledge on the path of gradients to get the minimum of our loss function. Although gradient improvement uses our base model’s loss function as a proxy to minimize the overall model error [50].

A decision tree, train only one model on the dataset and use that for classification. We can try different parameters for a bit or increase the data, but still, we are still using a single model. Even if we create an ensemble, all the models are trained and separately applied to the dataset.

Instead of training all models in isolation from each other, successive train models are boosted, with each new model trained to correct the mistakes made by the previous ones. Models are added sequentially until there can be no further changes. The advantage of this iterative technique is that the new models added are focused on correcting the mistakes produced by other models.

We used the GridSearch strategy to find the final model. Following are the parameters used in GridSearch.

- minimum child weight =  1, 5, 10
- gamma = 0.5, 1, 1.5, 2, 5
- subsample = 0.6, 0.8, 1.0
- colsample bytree = 0.6, 0.8, 1.0
- maximum depth = 3, 4, 5

**Fig. 6:**
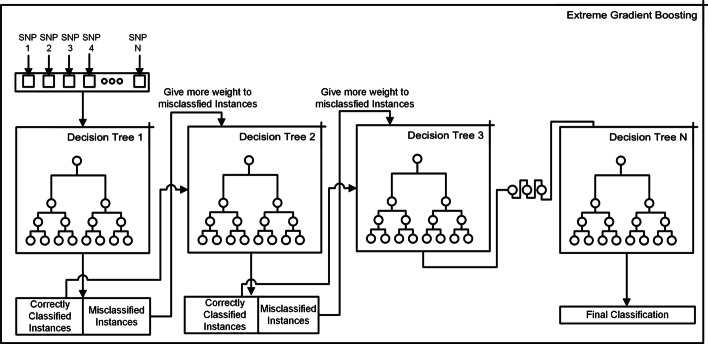
Extreme gradient boosting. XGBOOST trains models in succession, with each new model being trained to correct the errors made by the previous ones. Models are added sequentially until no further improvements can be made

Figure 6 shows the structure of an XGBOOST.

#### Ensembles of ANN and LSTM

Ensemble learning is the process by which multiple classifiers, are strategically generated and combined to solve a particular computational intelligence problem. Ensemble learning is primarily used to improve the prediction. As this article is focused on improving the accuracy of prediction not on finding the actual casual SNP so, an ensemble of different classifiers can be utilized [51, 52]. The important point here is which model will be used as a part of the ensemble. We can make decision based on the performance of the model on training data or validation data. Both training accuracy and validation accuracy can be combined to select the best model. We choose those models for which validation accuracy was greater than 0.92. The is no hard and fast rule for selecting the simple models because it also depends on the knowledge learned by each model. To find the models we tried all the combinations of the following parameters and threshold on validation accuracy. The rationale behind using this approach is each parameter given below can affect the performance of the model and also the knowledge learned by the model. To see that we analyzed the confusion matric of each model. As you can see in the figure some models performed well for Brown eyes and some for Blue-Green eyes. When we use a combination of all models then a well-defined boundary is plotted in hyperdimensional space which improves accuracy. There is no benchmarking for this process. If only those models are selected which have the same confusion matrix then the ensemble method will not improve the accuracy [53].

- Activation Function = Sigmoid, Relu, Softmax
- Dropout Rate = 0.2, 0.3, 0.5
- Optimizer = Adam, SGD, RMSprop
- Batch Size = 1, 10, 15, 20
- Validation Split = 0.2, 0.3, 0.4
- Number of Epochs = 10, 20, 50, 100

**Fig. 7:**
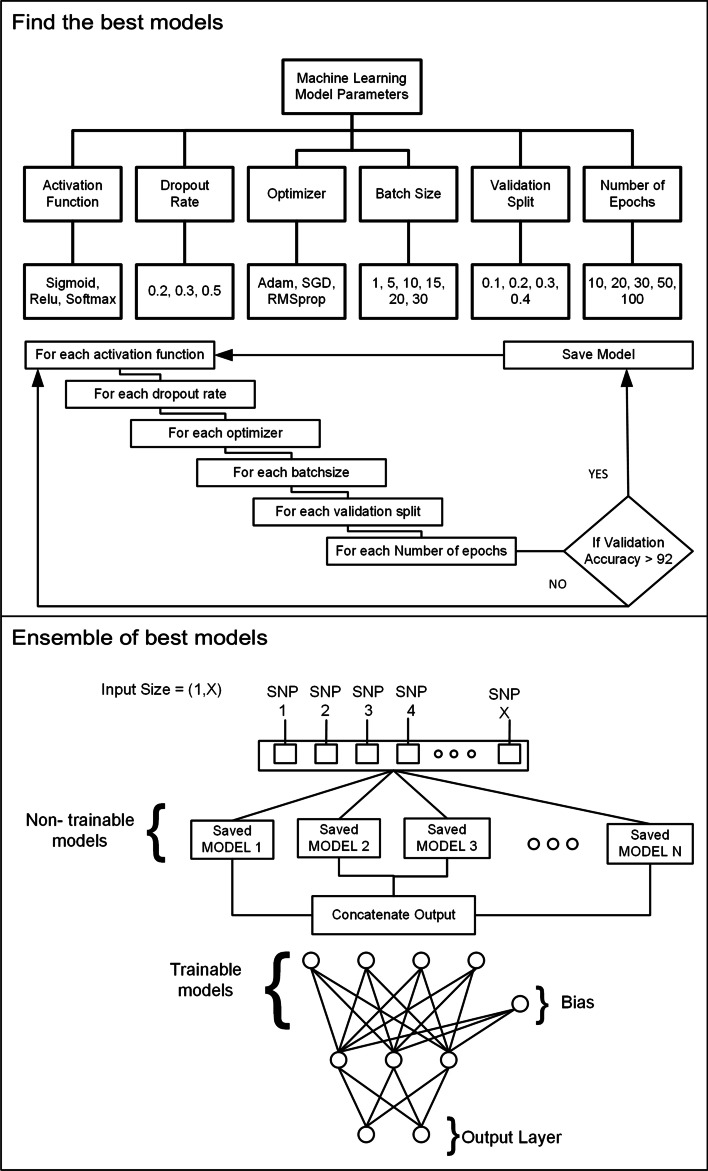
Ensemble approach. “Find the best models” show the approach to find the best model. Each combination of the different parameters is executed to find the best model. This is computationally expensive to find the models to be included in the ensemble. “Ensemble of best models” shows the final model. All the models which are to be used in the ensemble are non-trainable and their output is combined and connected with the fully connected network to produce the final model

Figure 7 shows the Ensemble approach for prediction.

## Results

Considering the different numbers of SNPs can strongly affect the performance of a model. Form a machine learning perspective SNPs are acting as features and considering more features will result in overfitting. For any phenotype finding the optimal SNPs for classification is an important task [54]. Moreover, the different SPNs are responsible for different phenotypes so, the selected SNPs which perform well for one particular phenotype may not work for any other phenotype. To predict any other phenotype SNPs pre-selection process has to be repeated.

We applied all algorithms in this order.

- Machine Learning (1. Random Forest 2. XGBOOST)
- Deep Learning (1. Artificial Neural Network 2. 1DCNN 3. GRU, LSTM, and BILSTM)
- Ensembles of ANN and LSTM

For the eye-color dataset, the existing techniques have an accuracy of about 90–96%. So, we applied algorithms to meet that accuracy. For type-2 diabetes we already got high accuracy, so we stopped at that Random Forest.

Table 5 summaries the results of Random Forest and Extreme Gradient boosting Classifier [55] for all the dataset containing a different number of SNPs, mentioned in the first row. We used a GridSearch for finding the optimal parameters for each model. All other cells are representing the Accuracy of the model for SNPs in the column and the classifier in a specific row.

For extreme gradient boosting we tried 3 loss functions which are hinge, logistic and logitraw, and boosters which are gbtree, gblinear, and dar. Extreme gradient boosting gave an accuracy of 0.93 when used with Logistic loss function [56] and default booster which is gbtree for both scaled and unscaled dataset.

**Table 5:** Results of random forest and extreme gradient boosting classifier

Classifiers	107	3	32	1560	9824	36,961	50,260	86,688
Random forest	0.92	0.89	0.91	0.90	0.86	0.81	0.81	0.82
(No scaling) (booster = gbtree, gblinear, dar)	0.88	0.89	0.88	0.92	0.92	0.92	0.92	–
	0.88	0.89	0.91	0.87	0.82	0.83	0.82	–
	0.88	0.89	0.88	0.92	0.92	0.92	0.92	–
(No scaling) (loss function = hinge, logistic, logitraw)	0.91	0.89	0.92	0.92	0.92	0.92	0.92	–
	0.92	0.89	0.92	0.93	0.92	0.92	0.93	–
	0.91	0.89	0.92	0.91	0.92	0.92	0.93	–
(Scaling) (booster = gbtree, gblinear, dar)	0.88	0.89	0.88	0.92	0.92	0.92	0.92	–
	0.85	0.89	0.90	0.86	0.78	0.78	0.73	–
	0.88	0.89	0.88	0.92	0.92	0.92	0.92	–
(Scaling) (loss function = hinge, logistic, logitraw)	0.91	0.89	0.9211	0.93	0.92	0.92	0.92	–
	0.92	0.89	0.92	0.93	0.92	0.92	0.93	–
	0.91	0.89	0.92	0.91	0.92	0.92	0.93	–

“–” means no results because of too long computation time. The different boosters used for XGBOOST are gbtree, gblinear, and dar. Different loss functions used for XGBOOST are hinge, logistic, and logitraw. The first row represents the number of SNPs used for Eye-color classification. The first column represents the classifier and different boosters used for the model. Scaling and No Scaling means dataset is scaled or not scaled for particular experiment or not

Tables 6, 7, 8, and 9 show the results of ANN, GRU, LSTM, BILSTM, and 1DCNN with different parameters for datasets containing 3, 32, 107, and 1560 SNPs respectively. For each table from 6 to 9 the first column represents the model name and the first represents the different parameters used. The last column represents the Accuracy of a specific model with specific parameter values. Even a good architecture can perform badly when hyper-parameters are not tunned very well, so to find the optimal parameters we must search through a list of different parameters.

**Table 6:** Table summarizes the accuracy of ANN, GRU, BILSTM, LSTM, and 1DCNN model for 3 SNPs

Model, SNPs = 3	Activation	Dropout	Optimizer	Batchsize	Epochs	Validation	Accuracy
ANN	Sigmoid	0.2	Adam	1	10	0.2	0.88
	Sigmoid	0.2	Adam	10	20	0.4	0.89
	Relu	0.3	RMSprop	15	50	0.3	0.89
	Relu	0.3	SGD	1	50	0.2	0.89
GRU	Sigmoid	0.2	Adam	1	10	0.2	0.895
	Sigmoid	0.2	RMSprop	10	50	0.3	0.895
BILTM	Sigmoid	0.2	Adam	1	10	0.2	0.895
	Sigmoid	0.3	RMSprop	10	100	0.3	0.895
LSTM	Sigmoid	0.2	Adam	1	10	0.2	0.9
1DCNN	Sigmoid	0.2	Adam	1	20	0.2	0.88
	Sigmoid	0.2	Adam	1	50	0.2	0.89
	Softmax	0.3	RMSprop	20	100	0.3	0.89
	Softmax	0.2	Adam	20	50	0.2	0.89
	Relu	0.3	RMSprop	15	50	0.4	0.89

LSTM performs well with an accuracy of 0.9%

**Table 7:** Table summarizes the accuracy of ANN, GRU, BILSTM, LSTM, and 1DCNN model for 32 SNPs

Model, SNPs = 32	Activation	Dropout	Optimizer	Batchsize	Epochs	Validation	Accuracy
ANN	Softmax	0.3	RMSprop	10	100	0.2	0.91
	Softmax	0.3	SGD	1	100	0.2	0.91
	Relu	0.3	RMSprop	15	50	0.2	0.91
	Relu	0.3	SGD	20	100	0.2	0.92
	Relu	0.3	SGD	1	100	0.3	0.92
GRU	Sigmoid	0.2	Adam	1	10	0.2	0.92
	Sigmoid	0.3	Adam	1	50	0.2	0.91
	Sigmoid	0.2	SGD	1	20	0.2	0.91
BILSTM	Sigmoid	0.2	RMSprop	15	20	0.2	0.91
1DCNN	Sigmoid	0.2	Adam	1	20	0.3	0.92
	Sigmoid	0.2	Adam	15	100	0.3	0.92
	Softmax	0.3	Adam	10	50	0.2	0.91
	Softmax	0.2	RMSprop	20	100	0.2	0.91
	Relu	0.3	RMSprop	10	50	0.2	0.91

ANN, GRU, and 1DCNN perform well with an accuracy of 0.92%

**Table 8:** Table summarizes the accuracy of ANN, GRU, BILSTM, LSTM, and 1DCNN model for 107 SNPs

Model, SNPs = 107	Activation	Dropout	Optimizer	Batchsize	Epochs	Validation	Accuracy
ANN	Sigmoid	0.3	SGD	1	50	0.3	0.9
	Relu	0.2	SGD	1	10	0.3	0.9
	Softmax	0.2	Adam	1	10	0.2	0.91
	Relu	0.3	SGD	15	50	0.2	0.91
LSTM	Relu	0.2	SGD	10	50	0.2	0.9
	Relu	0.3	Adam	1	20	0.2	0.9
	Relu	0.3	SGD	1	50	0.2	0.9
	Sigmoid	0.2	SGD	1	10	0.4	0.91
1DCNN	Sigmoid	0.2	Adam	1	20	0.3	0.92
	Sigmoid	0.2	Adam	1	50	0.3	0.91
	Relu	0.3	Adam	1	20	0.2	0.895

1DCNN performs well with an accuracy of 0.92%

**Table 9:** Table summarizes the accuracy of ANN, GRU, BILSTM, LSTM, and 1DCNN model for 1560 SNPs

Model, SNPs = 1560	Activation	Dropout	Optimizer	Batchsize	Epochs	Validation	Accuracy
ANN	Sigmoid	0.2	Adam	1	10	0.3	0.94
	Sigmoid	0.2	Adam	1	100	0.2	0.93
	Sigmoid	0.2	SGD	10	100	0.2	0.945
	Relu	0.2	SGD	15	100	0.2	0.94
	Sigmoid	0.2	Adam	15	10	0.3	0.94
BILSTM	Sigmoid	0.2	SGD	1	100	0.1	0.93
	Sigmoid	0.2	SGD	1	30	0.2	0.94
GRU	Sigmoid	0.2	SGD	1	50	0.2	0.94
	Sigmoid	0.2	Adam	1	20	0.3	0.94
LSTM	Sigmoid	0.2	Adam	10	10	0.1	0.945
	Sigmoid	0.2	SGD	1	30	0.3	0.93
1DCNN	Relu	0.2	RMSprop	15	20	0.2	0.91
	Relu	0.2	RMSprop	20	50	0.3	0.9
	Relu	0.3	RMSprop	20	50	0.2	0.91
	Relu	0.5	RMSprop	20	50	0.1	0.91

ANN and LSTM perform well with an accuracy of 0.945%

**Fig. 8:**
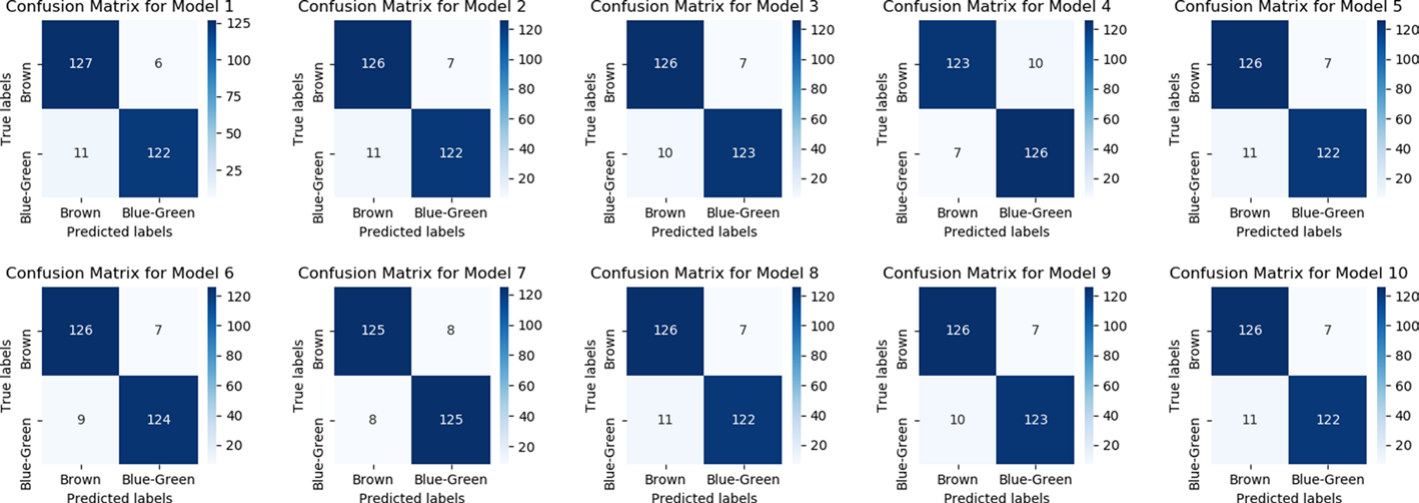
Confusion matrices of the 10 LSTM models used for the stacked ensemble model. There are few models that are good at classifying the Brown eyes and others at Blue-Green. Consider Model 3 which classifies Brown eyes very well, whereas model 4 performs well on Blue-Green. When results of such models are combined optimal result is obtained. There are few models that perform equally well for both classes like model 7

**Fig. 9:**
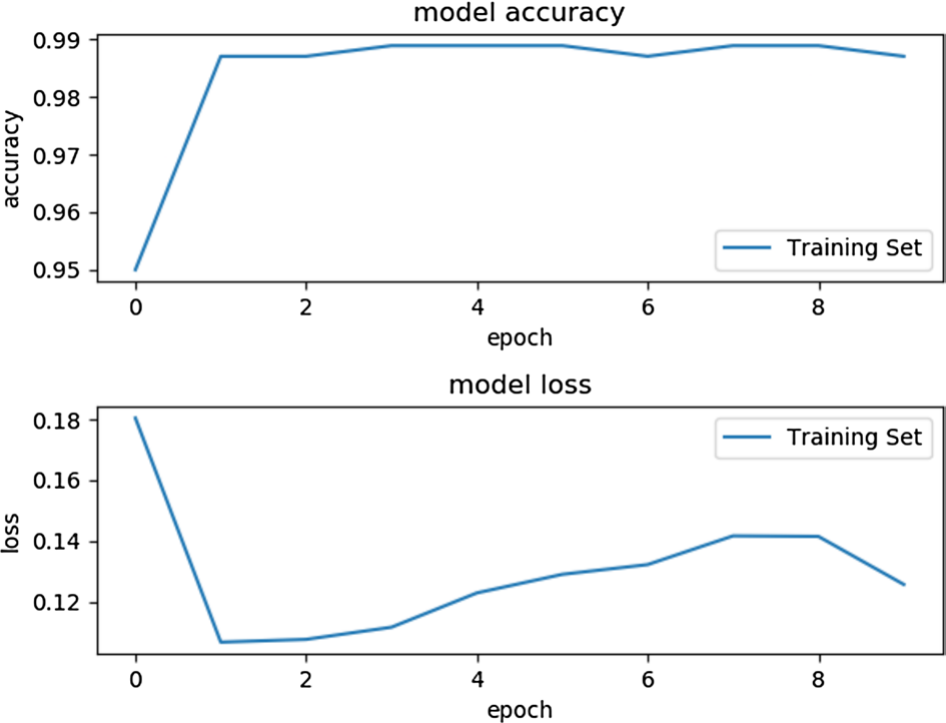
Accuracy and Loss of the best ensemble of LSTM for training. The final stacked model is training for 10 Epochs to avoid overfitting. The first plot shows the model accuracy on training data and the second plot shows the model loss for training data

**Fig. 10:**
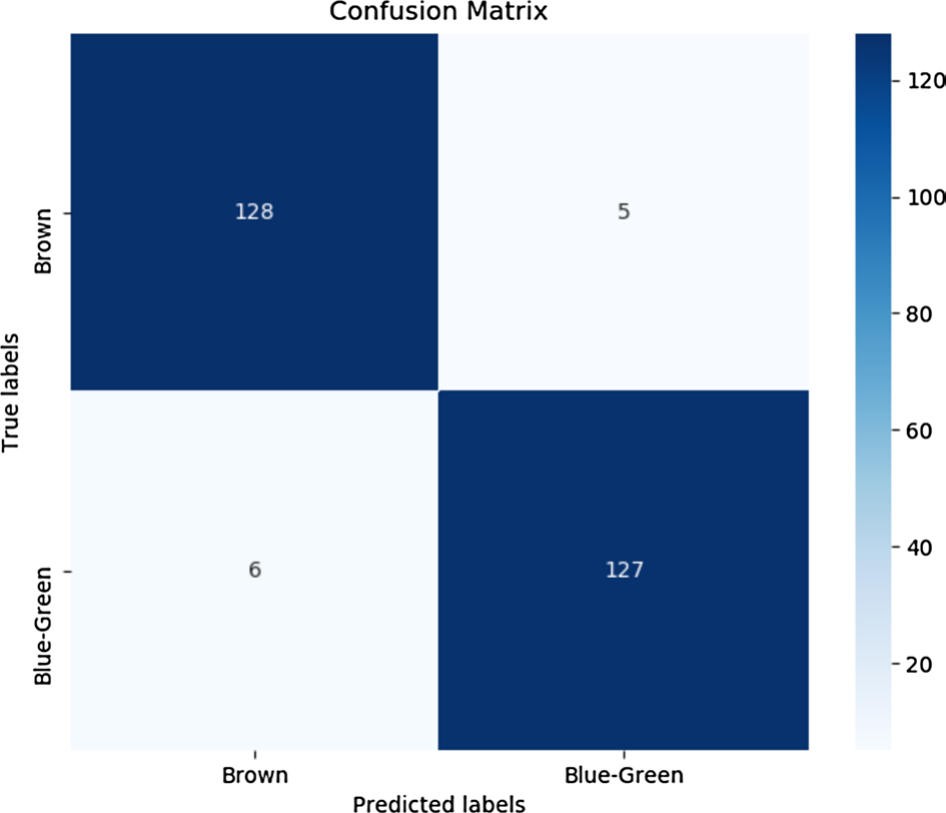
Confusion matrix of the best ensemble of LSTM

**Fig. 11:**
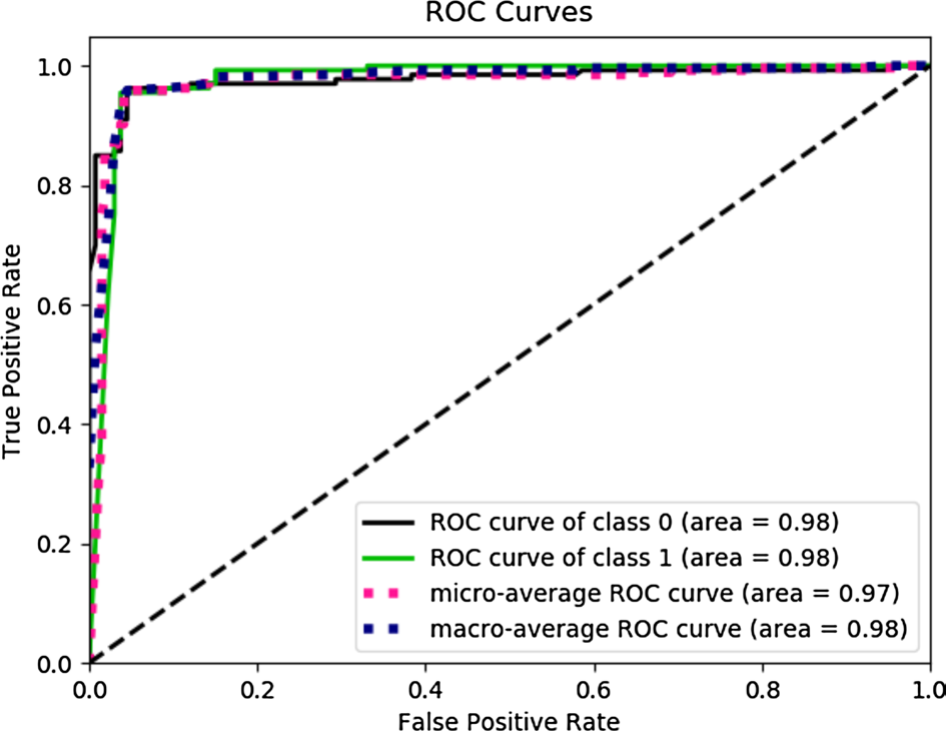
ROC of the best ensemble of LSTM. ROC for class 0 which is Brown eyes is 0.98, ROC for class 1 which is Blue-Green eyes is 0.98

In the end, we tried ensembles of LSTM and ANN. The table 10 shows the result for the Ensemble of ANN and LSTM. Figure 8 show the individual model confusion matrix used in the ensemble of LSTM, Figs. 9, 10 and 11 show the result of the final best LSTM based ensemble model.

**Table 10:** Ensemble of LSTM and ANN

SNPs = 1560	Accuracy
Ensemble of LSTM	0.96
Ensemble of ANN	0.95

10 LSTM models and 40 ANN models were used for prediction

### Type-2 diabetes prediction

**Fig. 12:**
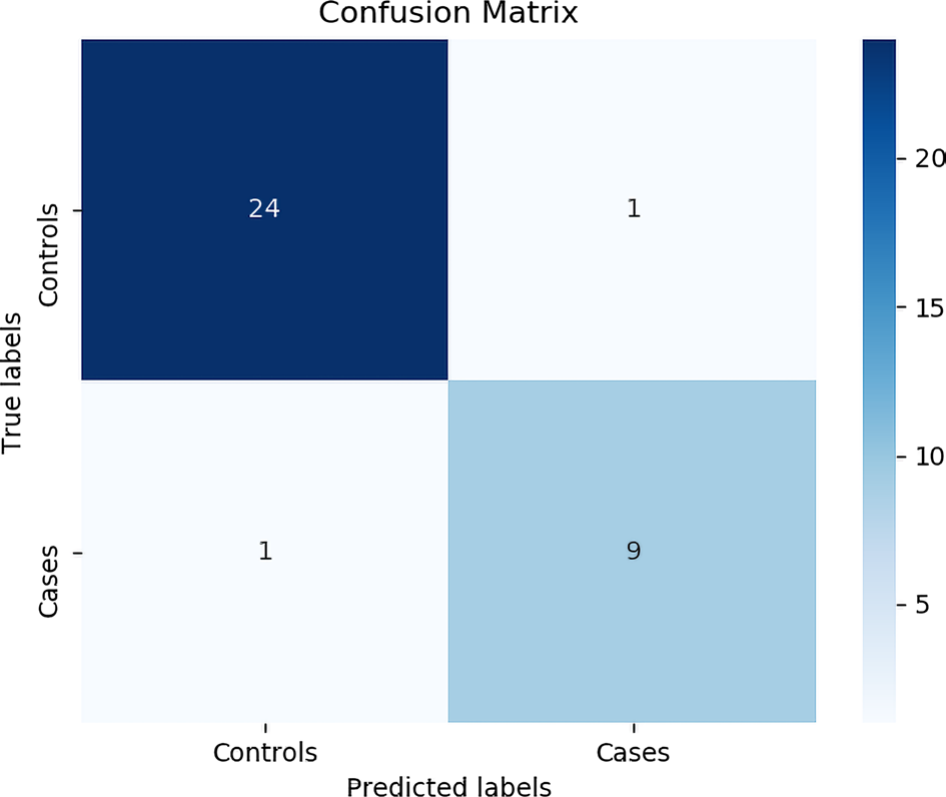
Confusion matrix of the best random forest model

**Fig. 13:**
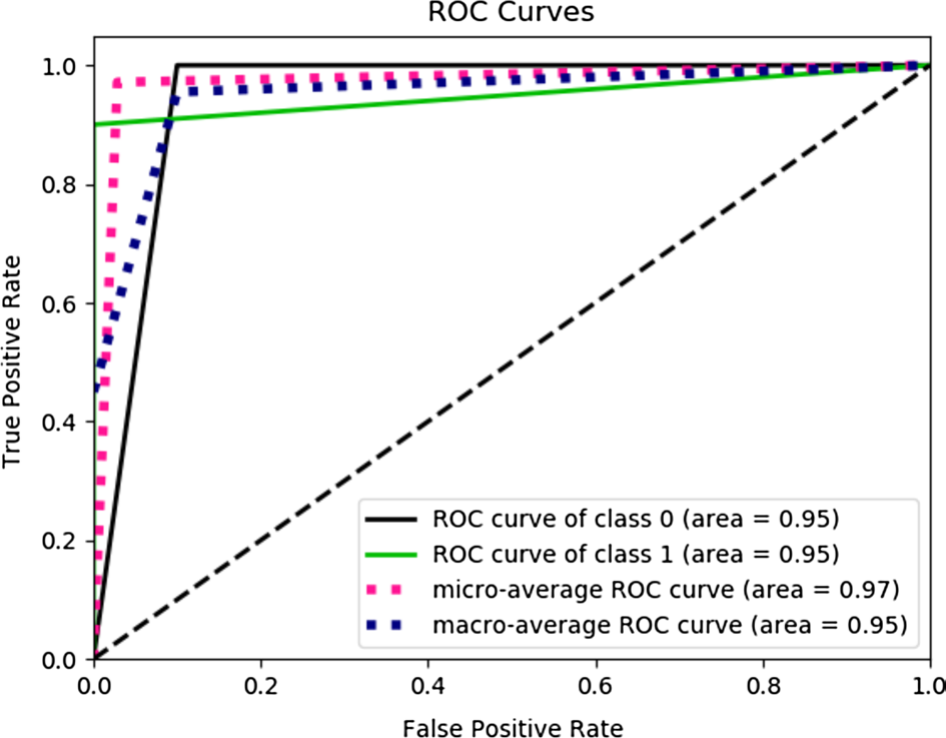
ROC for the best random forest model. ROC for class 0 which is controls is 0.95, ROC for class 1 which is Cases is 0.95

We also tested the proposed approach onType-2 diabetes phenotype. We considered different linear thresholds and the results for the optimal number of SNPs are summarized in table 11. Figures 12 and 13 shows the Confusion matrix and AUC of the best classification results.

- Total people = 104, Cases = 30, Controls = 74
- Training data split, Cases = 20 and Controls = 49
- Test data split, Cases = 10 and Controls = 25

**Table 11:** Type-2 diabetes results

SNPs = 32	Train accuracy	Test accuracy
Random Forest	0.98	0.97

Random forest with grid search was used for prediction

## Conclusion

Genotype–phenotype predictions are very useful especially in forensic. These predictions can help to identify SNP variant association with traits and diseases. Provide insight into the ethnic variation of complex traits. It leads to the discovery of novel biological mechanisms. To translate biological insights into medical advancements and making drugs. A combination of both statistical and Machine Learning approach for Genotype–phenotype predictions can yield the best results. Selecting SPNs based on mutation difference and the parameters used for the machine learning model can significantly impact the performance of prediction. Given more datasets, machine learning model predictions can be increased. Moreover, the non-linearity in the Machine learning model and the combination of SNPs Mutations while training the model increases the prediction. We considered binary classification problems but this approach can be extended to multi-class classification. Crime investigation can be assisted using the prediction of an individual’s externally visible characteristics (EVCs) like their eye, hair, and skin color from a crime scene stain.

Following are the specs of the computer and library used for implementing models and generating results. The system specifications are: Intel(R) Core(TM) 7-9750H CPU @ 2.60Hz, 16 GB RAM as well as a NVIDIA GeForce RTX 2060 GPU, running Microsoft Windows 10. Moreover, the development specifications are: Cuda compilation tools release 10.0, V10.0.130, Deep Learning framework Keras 2.4.3, Python 3.6.8, and Tensorflow 2.3.1. For eXtreme Gradient Boosting (XGBOOST) we used xgboost python library, version 1.0.2.

## Data Availability

All the data considered for this study is available at OPENSNP https://opensnp.org/.
