# Correction to: Eye‑color and Type‑2 diabetes phenotype prediction from genotype data using deep learning methods

**DOI:** 10.1186/s12859-021-04218-0

**Published:** 2021-06-11

**Authors:** Muhammad Muneeb, Andreas Henschel

**Affiliations:** Department of Electrical Engineering and Computer Science, Center for Biotechnology Khalifa University, Khalifa University of Science and Technology, Abu Dhabi, United Arab Emirates

## Correction to: BMC Bioinformatics (2021) 22:198 https://doi.org/10.1186/s12859-021-04077-9

Following the publication of the original article [1], errors were identified in the affiliation and funding note.

The changes have been highlighted in **bold typeface**.

Affiliation:

Department of Electrical Engineering and Computer Science, **Center for Biotechnology Khalifa University**, Khalifa University of Science and Technology, Abu Dhabi, United Arab Emirates.

Funding note:

The study is funded by Khalifa University Collaborative Internal Research Award CIRA-2019-076.

The original article [1] has been corrected.
